# Feasibility study of TSPO quantification with [^18^F]FEPPA using population-based input function

**DOI:** 10.1371/journal.pone.0177785

**Published:** 2017-05-17

**Authors:** Rostom Mabrouk, Antonio P. Strafella, Dunja Knezevic, Christine Ghadery, Romina Mizrahi, Avideh Gharehgazlou, Yuko Koshimori, Sylvain Houle, Pablo Rusjan

**Affiliations:** 1 Research Imaging Centre, Centre for Addiction and Mental Health (CAMH), Toronto, Ontario, Canada; 2 Morton and Gloria Shulman Movement Disorder Unit, E.J. Safra Parkinson Disease Program, Toronto Western Hospital, UHN, University of Toronto, Toronto, Canada; 3 Division of Brain, Imaging and Behaviour, Systems Neuroscience, Krembil Research Institute, UHN, University of Toronto, Toronto, Ontario, Canada; 4 Department of Psychiatry, University of Toronto, Toronto, Ontario, Canada; Biomedical Research Foundation, UNITED STATES

## Abstract

**Purpose:**

The input function (IF) is a core element in the quantification of Translocator protein 18 kDa with positron emission tomography (PET), as no suitable reference region with negligible binding has been identified. Arterial blood sampling is indeed needed to create the IF (ASIF). In the present manuscript we study individualization of a population based input function (PBIF) with a single arterial manual sample to estimate total distribution volume (*V*_*T*_) for [^18^F]FEPPA and to replicate previously published clinical studies in which the ASIF was used.

**Methods:**

The data of 3 previous [^18^F]FEPPA studies (39 of healthy controls (HC), 16 patients with Parkinson’s disease (PD) and 18 with Alzheimer’s disease (AD)) was reanalyzed with the new approach. PBIF was used with the Logan graphical analysis (GA) neglecting the vascular contribution to estimate *V*_*T*_. Time of linearization of the GA was determined with the maximum error criteria. The optimal calibration of the PBIF was determined based on the area under the curve (AUC) of the IF and the agreement range of *V*_*T*_ between methods. The shape of the IF between groups was studied while taking into account genotyping of the polymorphism (rs6971).

**Results:**

PBIF scaled with a single value of activity due to unmetabolized radioligand in arterial plasma, calculated as the average of a sample taken at 60 min and a sample taken at 90 min post-injection, yielded a good interval of agreement between methods and optimized the area under the curve of IF. In HC, gray matter *V*_*T*_s estimated by PBIF highly correlated with those using the standard method (r^2^ = 0.82, p = 0.0001). Bland-Altman plots revealed PBIF slightly underestimates (~1 mL/cm^3^) *V*_*T*_ calculated by ASIF (including a vascular contribution). It was verified that the AUC of the ASIF were independent of genotype and disease (HC, PD, and AD). Previous clinical results were replicated using PBIF but with lower statistical power.

**Conclusion:**

A single arterial blood sample taken 75 minute post-injection contains enough information to individualize the IF in the groups of subjects studied; however, the higher variability produced requires an increase in sample size to reach the same effect size.

## Introduction

Translocator protein 18 kDa (TSPO), described formerly as the peripheral benzodiazepine receptor (PBR), is widely recognized as a significant target for imaging by positron emission tomography (PET). The expression of this protein in microglia plays a significant role as an inflammation indicator following brain injury and neurodegenerative diseases[1]. Many radioligands have been developed to bind with TSPO *i*.*e*. [^11^C]PK11195, [^11^C]PBR28, [^11^C]DPA713, [^18^F]PBR111 and [^18^F]FEPPA. Since there is no region free of TSPO in the brain, the quantification of these radioligands requires an arterial input function (IF) to quantify the TSPO binding. Arterial sampling, which is needed to create the IF, requires arterial cannulation of the subject. This procedure may cause potential discomfort and discourage subjects from participating in PET studies. Tissue reference input function was used to quantify activated microglia using [^11^C]PK11195 tracer [2, 3]. Both reference and target tissue were extracted from PET data using an unsupervised clustering method. Another simplified method that has been used includes the use of the cerebellum as a pseudo-reference region in [^11^C]PBR28 PET data of Alzheimer disease [3].

In the present study, we have used [^18^F]FEPPA to quantify TSPO. We have previously demonstrated that the total distribution volume (*V*_*T*_) estimated with a two tissue compartment model (2-TCM) is the optimal parameter for quantifying TSPO using [^18^F]FEPPA [4], and that [^18^F]FEPPA binding, like any other second generation radioligand, is strongly affected by a single nucleotide polymorphism in the TSPO gene (rs6971) [5, 6]. In addition, in clinical studies investigating neuroinflammation, we have demonstrated that [^18^F]FEPPA *V*_*T*_ was higher in patients with Alzheimer’s disease compared to matched controls [7], however, no differences were found between patients with Parkinson’s disease and matched controls [8].

In a few instances, investigators have been able to overcome the issues regarding full arterial sampling by using different techniques based on image-derived techniques, models of input function and population-based input functions (PBIF) [9–11]. The image-derived techniques consist of: image derived input functions (IDIF) [10, 12] and image derived reference region time activity curve (TAC)[2]. IDIF allows a non-invasive measurement of blood tracer concentration directly from individualized dynamic images by using structures such as the blood pool in heart studies [13] and the internal carotid in brain studies [14]. Unlike full arterial sampling, the IDIF is not affected by delay and dispersion. However, in practice a few physical and physiological constraints limit the reliability of the IDIF technique [15]. For instance, PET cameras cannot always distinguish between the parent compound and the radio-metabolites. Therefore, one or a few blood samples are still necessary in order to be able to take into account metabolization and to scale the IDIF [16].

PBIF is a more simple method compared to IDIF, and it is obtained in two steps: 1) an average IF shape is calculated through a population of subjects who underwent the same protocol of injection, and 2) a study-specific scale calibrates the specific input function. PBIF is suitable for tracers whose individual IF shape behaves similarly to the population average IF and differs merely in amplitude. Specifically, this behavior has been widely validated in the quantification of cerebral metabolic rate of glucose using [^18^F]FDG data [17–19]. This finding has been extended to analyse a large array of tracers [20, 21].

Usually PBIF is applied more successfully using linearized methods (like Logan analysis) than using nonlinear compartmental models (for instance, 2-TCM). This is due to the fact that Logan analysis requires the area under the curve of the IF for each time frame, rather than the detailed temporal evolution of the IF[20] [22]. PBIF has a notable superiority to IDIF as it is not affected by the ambiguity between the parent tracer and its radio-metabolites, nor does it suffer from image quality issues such as the partial volume effect, both of which affect kinetic parameter quantification.

In the present study, we have investigated whether PBIF is applicable to quantify [^18^F]FEPPA *V*_*T*_ by 1) exploring differences in IF across 3 populations (healthy controls (HC), Alzheimer’s disease (AD) and Parkinson’s disease (PD)); 2) finding the optimal calibration for the PBIF and 3) confirming that by using the *V*_*T*_ estimated by the PBIF we can replicate the results from our previously published clinical studies that utilized ASIF.

## Material and methods

### Subjects and experimental design

The protocols of each study were approved by the corresponding institution’s Research Ethics Boards. Healthy Controls Study: The protocol was approved by the Center for Addiction and Mental Health Ethics Review Board and conformed to the Declaration of Helsinki. Alzheimer’s disease study: The protocol was approved by the Research Ethic Boards of Centre for Addiction and Mental Health and Baycrest Health Sciences, and conformed to the Declaration of Helsinki. Parkinson’s disease study: The study was approved by the Centre for Addiction and Mental Health Research Ethics Board and the University Health Network Research Ethics Board, and conformed to the Declaration of Helsinki. Informed consent: All subjects provided a written informed consent after all study procedures were fully explained. For participants with AD, a written consent was also obtained from the subject’s primary caregiver or substitute decision maker.

[^18^F]FEPPA affinity is known to be sensitive to the rs6971 single nucleotide polymorphism in the TSPO gene. Therefore, the population is divided based on binding categories: high affinity binders (HABs) (subjects carrying only the Ala147 allele), low affinity binders (LABs) (subjects carrying only the Thr147 allele) and mixed affinity binders (MABs). The TACs (before and after partial volume effect correction) and the ASIF derived-input functions used in the present work are from the previously published studies. In total we have reanalyzed 39 HC, 18 patients with AD and 16 patients with PD [4, 7, 8].

[^18^F]FEPPA was synthesised following Wilson *et al* [23]. For all the experiments, 5±0.5 mCi of [^18^F]FEPPA was injected manually as a quick bolus and was followed by flushing 10–15 ml of saline solution (total time <10 seconds). Each subject underwent a 2-hour PET scan after [^18^F]FEPPA injection, using a high-resolution research tomograph (HRRT) (CPS/Siemens, Knoxville, TN, USA). Images were reconstructed using filtered-back projection (FBP) with a Hann filter cut off at the Nyquist frequency [24]. The first frame (background frame) was of variable length depending on the time between the start of acquisition and the signal recorded in the field of view (FOV). The subsequent image frames were defined as 5×30, 1×45, 2×60, 1×90, 1×120, 1×210, and 22×300 seconds. Each volume in the reconstructed images has 256×256×207 cubic voxels measuring 1.22×1.22×1.22 mm^3^. Images were decay corrected to the scan starting time (*i*.*e*. the beginning of the background frame). Regions of interest (ROIs) were delineated by proton-density MRI images anatomically using an in-house software, ROMI[25]. ROMI fits a standard template of ROIs to an individual high-resolution MRI based on the probability of gray matter, white matter, and cerebrospinal fluid. The individual magnetic resonance image with ROIs properly superimposed are then co-registered to the summed [^18^F]FEPPA PET image using a mutual information algorithm. TACs were generated by masking the dynamical PET images with the re-sliced ROIs.

The same ROIs were reproduced from the previous studies. *V*_*T*_ values assessed in these ROIs were estimated separately from left and right and later averaged. Please refer to the aforementioned publications for further details regarding polymorphism genotyping, magnetic resonance image acquisition, PET and ROI delineation.

### Arterial blood acquisition and analysis for standard input function (ASIF) generation

An automatic blood sampling system (ABSS, Model # PBS-101 from Veenstra Instruments, Netherlands) continuously counted radioactivity in arterial blood at a rate of 150 ml/hour for the first 22.25 minutes post [^18^F]FEPPA-injection. In addition, 4 to 10 ml arterial blood manual samples were taken at 2.5, 12, 15, 30, 45, 60, 90 and 120 min post-injection. An aliquot of each blood sample was taken to measure radioactivity concentration in total blood using a Packard Cobra II gamma counter cross-calibrated with the PET system. The remaining blood was centrifuged (1500g, 5 min) and a plasma aliquot counted. A bi-exponential interpolation of the blood-to-plasma ratio was used to convert the blood TAC measured by automatic sampling into the plasma radioactivity curve. The remaining volume of plasma of each manual sample (except the one at 15 minutes) was used to determine the fraction of metabolites in plasma using an HPLC method of analysis[26]. A hill function (1 –(*β*.*t*^*δ*^/(*t*^*δ*^ + *α*))) was used to fit the fraction of unmetabolized tracer and to create the input function (unmetabolized parent in plasma). Delay and dispersion were estimated from the trues in the FOV, as described previously [4].

### Population based input function (PBIF) generation

Arterial input functions measured from 24 randomly selected subjects (8 healthy controls, 8 PD and 8 AD patients, S2 File) were used to generate the PBIF according to the following steps: 1) the concentration of parent compound in plasma (decay, delay and dispersion corrected) was expressed in SUV units normalizing by amount of injected radiotracer divided by subject weight, 2) all individual SUV-IFs were shifted slightly in time to start simultaneously, 3) individual parent compounds were then averaged together to form a standard PBIF, 4) subject-specific PBIFs were individually scaled (“calibrated”) using the radioactivity of unmetabolized radioligand in plasma sampled at times 12, 30, 45, 60, and 90 min post-injection in addition to 2 pseudo-times 52 and 75 min calculated as the average of plasma sampled at 45 and 60 min, and 60 and 90 min, respectively, 5) for each subject, the standard PBIF was delayed and decay corrected to account for the background frame of variable length of the PET image. Results were analyzed as a function of the time-plasma sample used to rescale the PBIF.

### Kinetic modeling

*V*_*T*_ estimated with ASIF was calculated using the 2-TCM as described in our previous work[4]. *V*_*T*_ estimated with PBIF was calculated using Logan graphical analysis (GA)[27]:

$$\frac{\int_{0}^{t}C\left(\tau \right)d\tau }{C\left(t\right)}=V_{T}\frac{\int_{0}^{t}C_{p}\left(\tau \right)d\tau }{C\left(t\right)}+intercept\;(for\;t>t^{*}),$$ (1)

where *C*(*t*) is the [^18^F]FEPPA regional TACs, *C*_*p*_(*t*) the input function and t* the time in which the plot reached linearity. t* was determined using the maximum admissible error criterion as implemented PMOD (PMOD group, Zurich, Switzerland) and described in Ichise *et al*[28]. This method automatically searches for the minimum time after which every data point in the Logan plot shows a relative error (defined as (measured-predicted)/predicted) lower than a given threshold (10% was used). Note that the vascular contribution in GA was dismissed, as it would require having a blood TAC for the PBIF, implying an overestimation of *V*_*T*_. The *V*_*T*_ values derived from GA highly correlated with the 2-TCM applied to the same cohort of healthy subjects and patients in HABs and MABs groups (R^2^ = 0.82, p<0.001 Figure B in S1 File).

### Statistical analysis

The optimal “calibration” of the PBIF was determined by minimizing the area under curves at the end of scan (AUC (t = 120min)) respect of the ASIF. ANOVA analyses were used to compare between AUC means of the 3 groups. Bland-Altman (B&A) plots and Pearson correlation coefficients were used to evaluate the interchangeability, 95% limits of agreement (LoA, ranges within the 95% of the difference between method should lie[29]) and the strength of the relationship between *V*_*T*_ estimated with ASIF and PBIF. Intraclass Correlation Coefficient (ICC) was used to agreement of *V*_*T*_ between methods and it is defined as ICC = (BMS-WMS)/(BMS+(n-1)WMS), where BMS = the mean of summed square between subjects, WMS = mean of summed squares within subjects and n = number of subjects. The variability within an ROI was calculated as the standard deviation/mean of *V*_*T*_ values.

## Results

### Comparison between ASIF and PBIF

**Fig 1:**
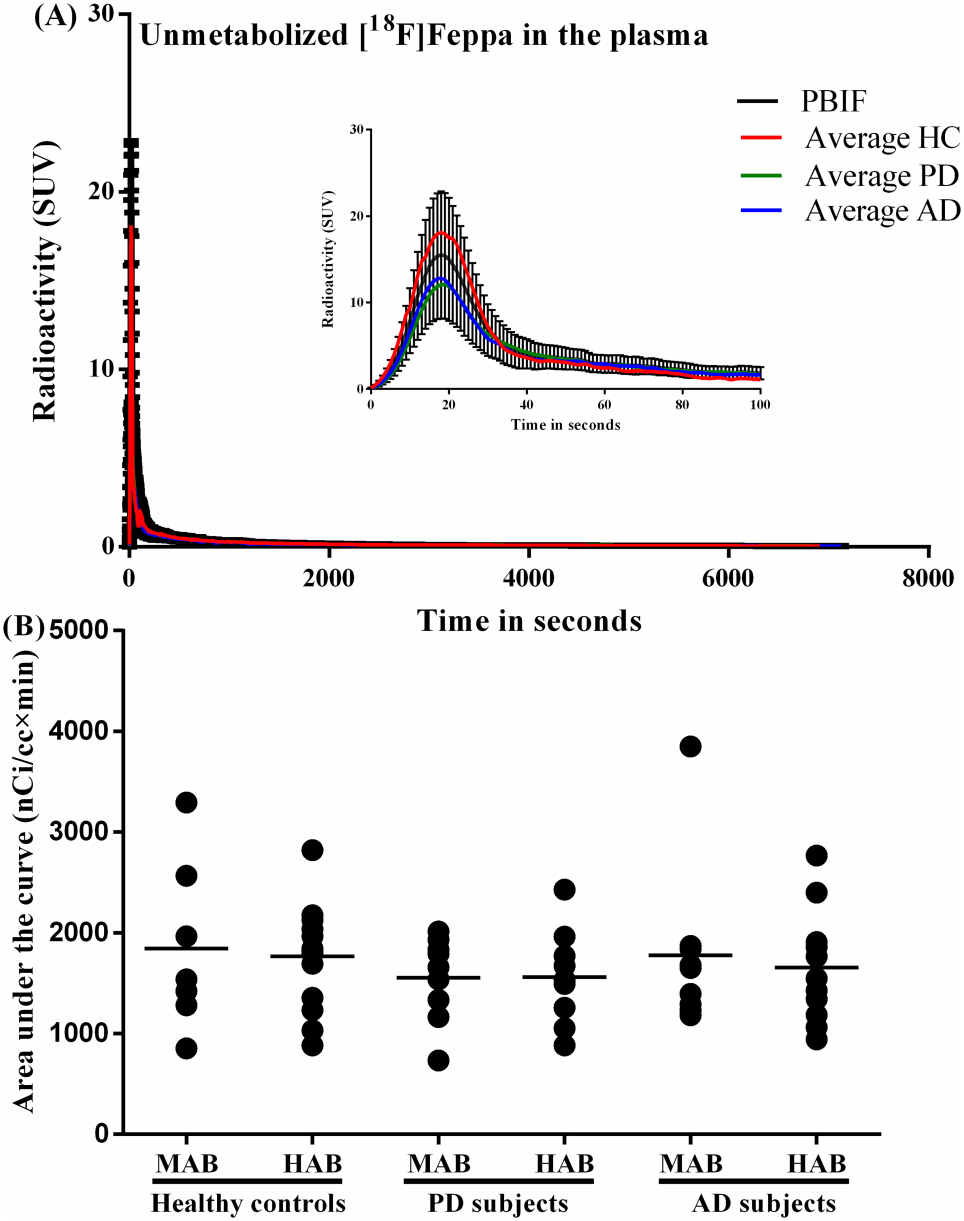
Population based input function. A) Average (n = 24) time evolution of unmetabolized [^18^F]FEPPA in plasma from HC (n = 8), PD (n = 8) and AD (n = 8) subjects. The inner plot shows details of the first 100 seconds post injection. B) The area under the curve of the input functions created from arterial blood sampling did not differ between 21 HC, 18 AD and 16 PD.

PBIF can be used successfully only in cases where the shape of the IF is not substantially different between groups or conditions. Thus, the first goal of the work was to examine whether such significant differences in the ASIF can be observed amongst the different conditions (*e*.*g*. control vs disease or MABs vs HABs). A visual inspection of the average IF for each group did not show important differences between groups (Fig 1A). The log-log plot similarly showed no important difference neither for the peak nor for the tail of the IF across groups (Figure A in S1 File). Using the AUC (t = 60 min) and the AUC (t = 120 min), a one-way ANOVA did not show statistically significant differences between groups (*F* (5, 18) = 1.07, *p* = .41 and *F* (5, 18) = 0.962, *p* = .47 respectively) (Fig 1B).

### Scaling and validation of PBIF in Healthy Controls (HC)

**Fig 2:**
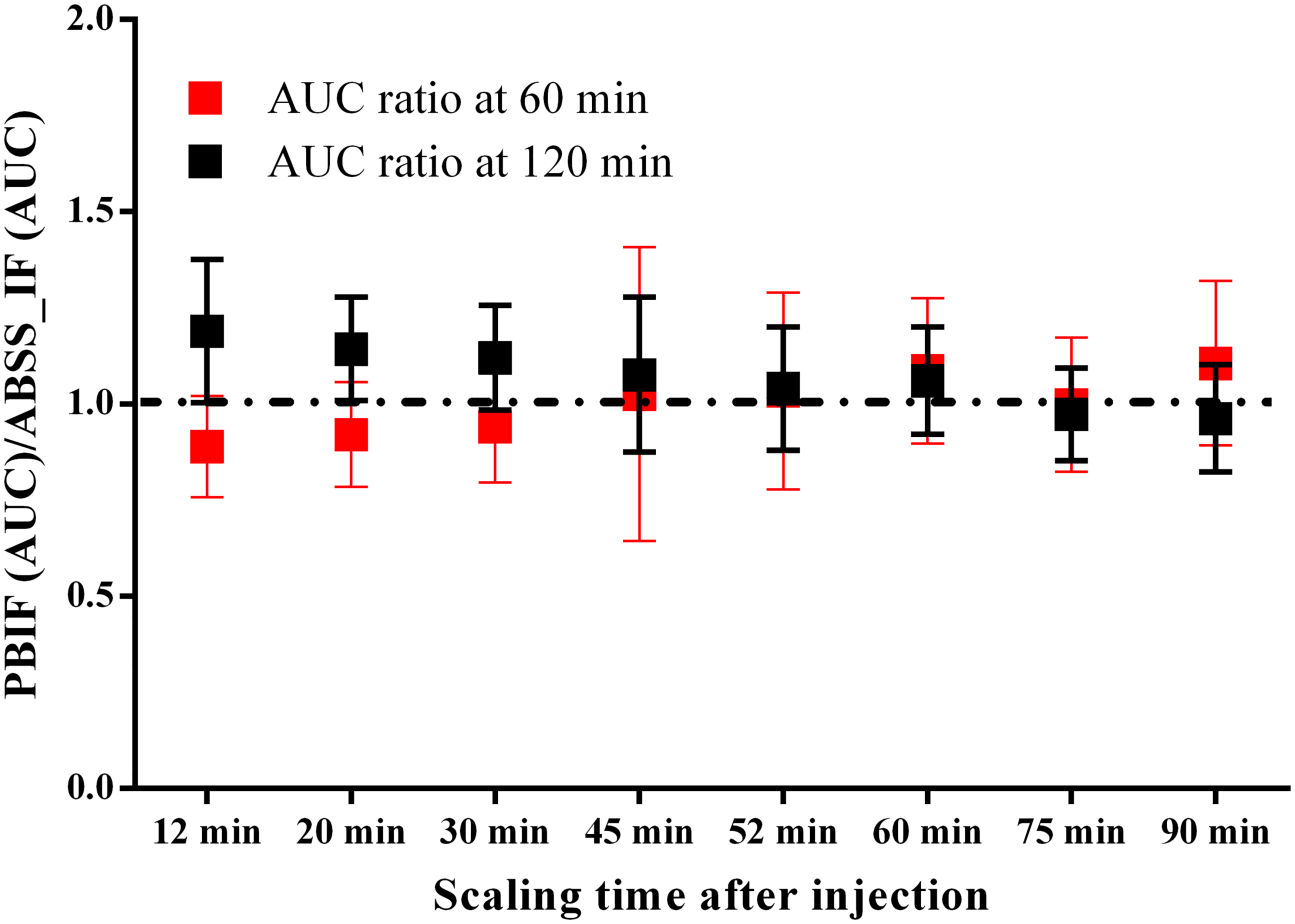
Comparison of the AUC of the PBIF and ASIF at 60 and 120 min as a function of the time-sample used to rescale. Samples at 75 min minimize the difference and SD. It should be noted that sample at 52 min is the average between 45and 60 min and 75 is the average between 60 and 90 min. the result is the average between 21 HC, 18 AD and 16 PD.

As shown in eq 1. GA depends on the average of the AUC(t) of IF in each time-frame. Therefore, the scaling of PBIF with different arterial samples was compared based on the AUC (t = 60 min) and the AUC (t = 120 min) with respect to ASIF for the different clinical populations. Overall, the scaling of the PBIF with samples taken between 60 and 90 min produced a similar AUC at 120 min to the ASIF. Hence, the 75 min sample optimized the variability across groups (Fig 2). In fact, the sample at 75 min is not a real sample, it is the average between the sample at 60 and 90 min. The decrease of variability can be due to cancelation of noise. Based on bias and variability of AUC ratio the sample at 75 min was chosen to calibrate the PBIF (PBIF75).

### Agreement of V_T_ between ASIF and PBIF75 and replication of published results

#### Healthy Controls (age range: 45–82 years)

**Fig 3:**
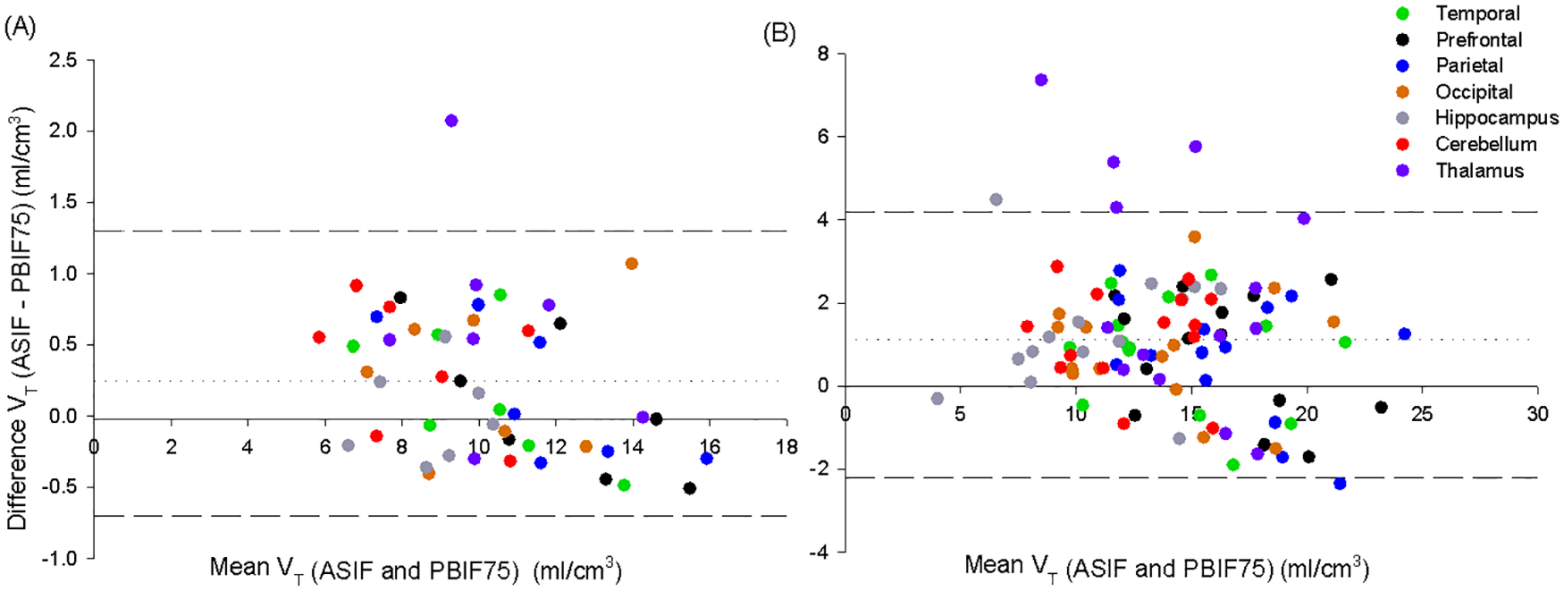
Bland-altman plot of *V*_*T*_ assessed by ASIF (2-TCM) and PBIF75 (Logan plot) for 7 regions of interest in healthy controls. FEPPA *V*_*T*_*s* derived by Logan PBIF75 plot slightly underestimated (Bias < 1 mL/cm^3^) those derived with 2-TCM. (A) In MABs (n = 7), the 95% limits of agreement (dashed *lines*) was between -0.7 to 1.3 mL/cm^3^ and no systematic bias was observed (r = 0.12, p = 0.6). (B) In HABs (n = 14), the 95% limits of agreement (dashed lines) was between -2.2 to 4.2 mL/cm^3^ and no systematic bias was observed (r = 0.08, p = 0.7).

The healthy controls discussed in this section are the same sample that will be used in the AD section below and have been previously reported in Suridjan *et al*[7]. TACs PVE corrected of the temporal, prefrontal, inferior parietal, occipital and cerebellar cortices, hippocampus and thalamus from 7 MABs and 14 HABs were used. *V*_*T*_ estimation using ASIF and PBIF75 correlated very well (i.e. r = 0.93, p<<0.001). B&A plot (Fig 3) showed a small bias (PBIF75 underestimated < 1 mL/cm^3^ the ASIF) and that the differences between methods did not vary in any systematic way over the range of measurement. The LoA depends on the ROIs. In HABs, it ranged from -2.2 to 4.2 mL/cm^3^ in large cortical areas and worsened for smaller areas. These values are relatively high given that *V*_*T*_ ranges between 8 and 25 mL/cm^3^ in cortical areas of HABs subject. The ICC for all the ROIs (except the thalamus) was >0.8 showing that the variability between methods is lower than between subjects. The ICC for HABs for the thalamus was 0.57, which means that variability between methods is similar to between subjects. Variability of *V*_*T*_ between subjects was smaller for ASIF (~24% in HABs) than in PBIF75 (~28% in HABs). It was studied that using the sample at 75 minutes reduced the B&A limits of agreements (Figure C in S1 File).

#### Genotype effect

**Fig 4:**
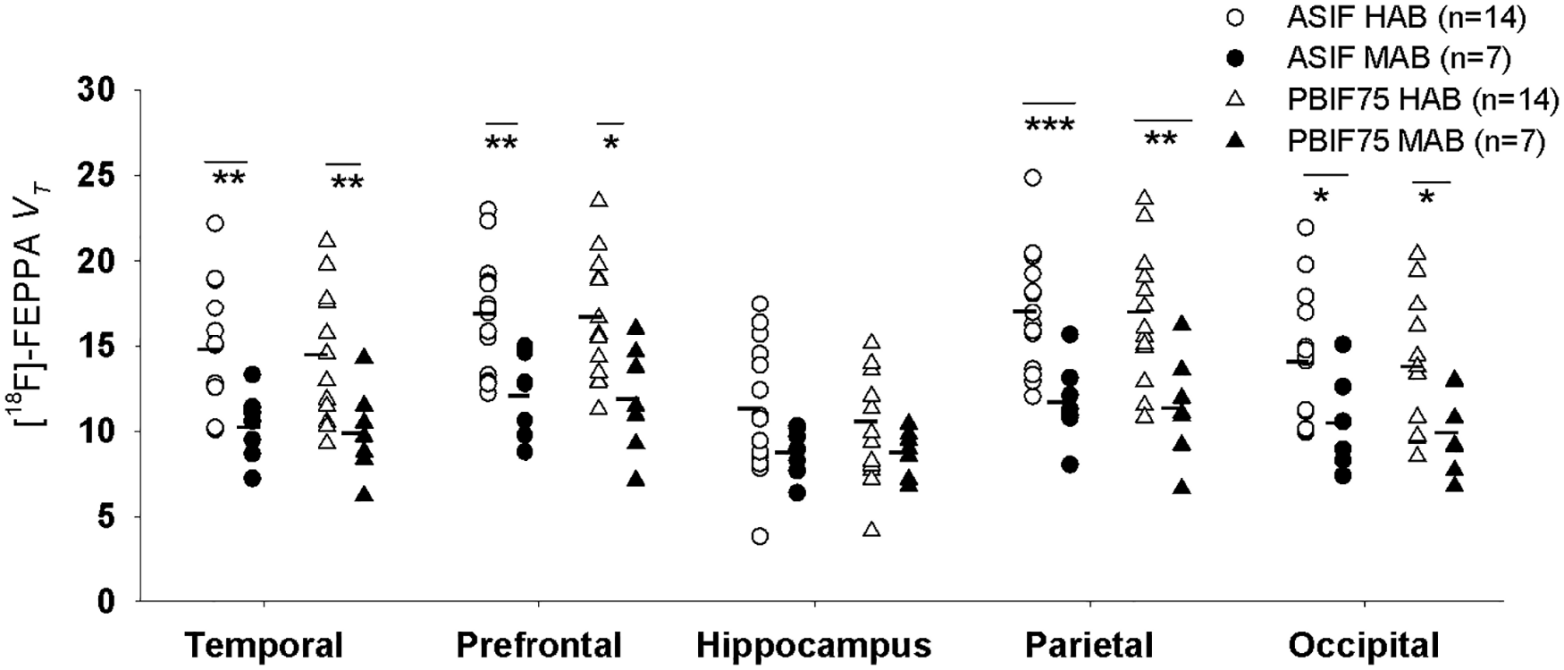
Total distribution volume (*V*_*T*_) calculated using ASIF (2-TCM) and PBIF75 (Logan plot). In Temporal, Prefrontal, Hippocampus, Parietal and Occipital regions stratified as HAB and MAB groups. ASIF showed an average reduction of 29% in *V*_*T*_ across ROIs while PBIF showed a reduction of 26%. 5/7 ROIs survived the multiple region Bonferroni adjustment using ASIF and only 2/7 passed the Bonferroni adjustment using PBIF75. Images were partial volume effect corrected prior to TAC extraction. * p< 0.05, ** p<0.01 *** p< 0.001.

We use PBIF75 and TACs corrected by PVE to replicate the previously observed effect of genotype (HABs vs MABs) on *V*_*T*_ of HC [7] (Fig 4). While ASIF showed an average reduction of 29% in *V*_*T*_ across ROIs, the reduction was 26% in PBIF75. Student t-test showed p< 0.03 for all the ROIs (with exception of the hippocampus) and on average p-values from PBIF75, were 72% higher than ASIF. In the hippocampus, the difference between MABs and HABs was not significant (ASIF p = 0.057 and PBIF75 p = 0.2). On other hand, while 5/7 ROIs would survive a Bonferroni correction with ASIF, only 2/7 would pass in PBIF75. These results are consistent with the increase in the between subjects variability in the PBIF75, which reduces the effect size in a given sample size.

#### Alzheimer’s disease study (AD)

Increased TSPO in subjects with AD compared to HC is a result consistently replicated in PET TSPO studies. Using [^18^F]FEPPA and ASIF we have found that, depending on the ROI, AD-patients (n = 10 HABS and 8 MABs) showed 20%-56% higher adjusted *V*_*T*_ than age-matched controls (n = 14 HABs and 7 MABS)[7]. In the present study, we used the same PVE-corrected TACs to: 1) validate the PBIF75 in AD patients, and 2) replicate the difference in *V*_*T*_ between groups.

**Validation of the PBIF75 in AD patients:** In total, 126 *V*_*T*_s (18 subjects, 7 ROIs) were calculated using PBIF75. The agreement of PBIF75 derived *V*_*T*_ and those estimated using ASIF was excellent in the temporal, prefrontal, inferior parietal, occipital and cerebellar cortex (ICC>0.9). B&P showed PBIF underestimated 1.5 ml/cm^3^ ASIF independently of the genotype and the LoA were ±4.6 ml/cm^3^ for HABs and ±3.2 ml/cm^3^ for MABs. The agreement in the thalamus (ICC = 0.76, bias = 4.1 ml/cm^3^, LoA = ±8.7 ml/cm^3^) and hippocampus (ICC = 0.5, bias = 6.1 ml/cm^3^, LoA = ±9.5 ml/cm^3^) in HABs was markedly lower driven by 2 “outliers” in the thalamus and 1 in the hippocampus. The *V*_*T*_ of “outliers” were strongly underestimated due to the maximum error criteria determined a very late t* which included only a few points (~5) in the linear regression. The thalamus (ICC = 0.89, bias = 2.4 ml/cm^3^, LoA = ±3.4 ml/cm^3^) and hippocampus (ICC = 0.82, bias = 2.5 ml/cm^3^, LoA = ±4.1 ml/cm^3^) in MABs showed more bias respect of the cortical ROIs.

**Fig 5:**
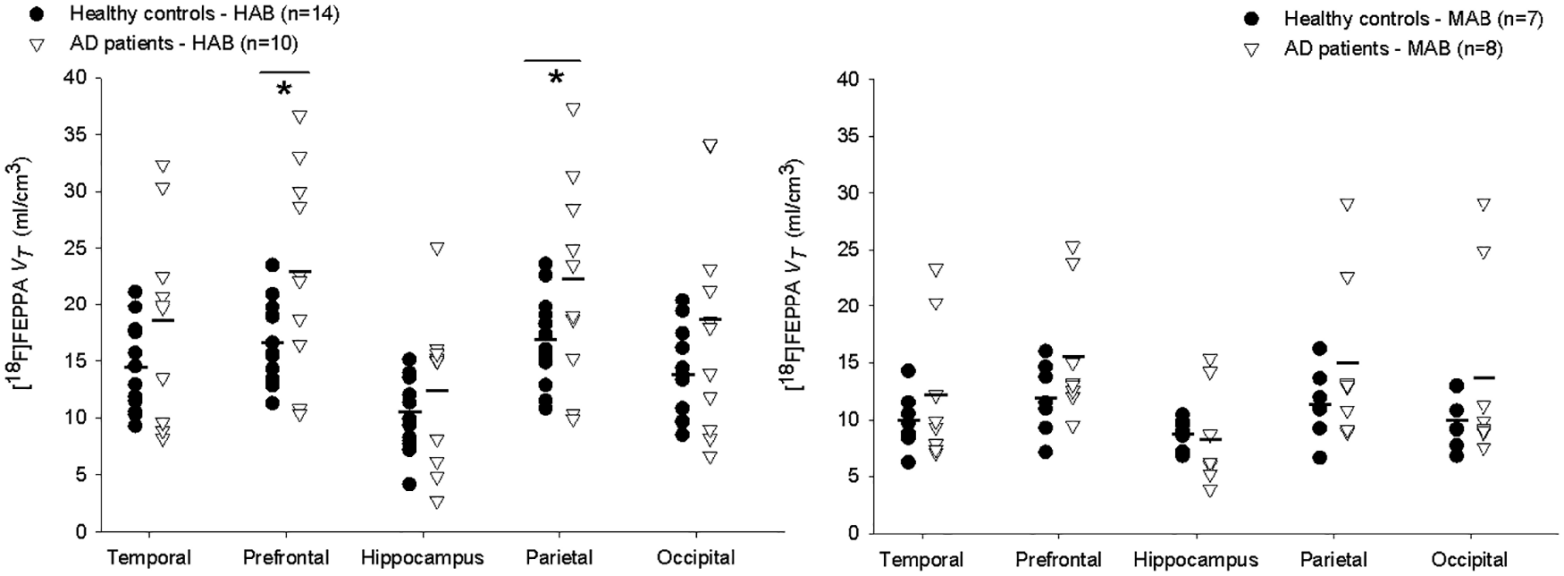
Comparison of [^18^F]FEPPA regional total distribution volume (*V*_*T*_) between healthy control subjects and AD patients calculated with PBIF75 for HABs (left) and MABs (right). (*cf*. figure 1 in ref [7]). PBIF75 estimation led to the same conclusion as the previously published with ASIF: [^18^F]FEPPA *V*_*T*_ in AD patients is on average 13–48% higher than HC (A Factorial ANOVA with genotype and age as covariate showed: p<0.05 in the Temporal, Prefrontal, Parietal and Occipital and p = 0.4 in the hippocampus). Images were partial volume effect corrected prior to TAC extraction. * p< 0.05 in ANOVA within group.

**Replication of the difference in *V***_***T***_ **between groups:** Overall, the factorial ANOVA with the PBIF75 *V*_*T*_ agrees with the previous work (Fig 5, Table A in S1 File): the adjusted *V*_*T*_ in AD was between 13% and 48% higher than in HC; however, the p-values were sensibly higher than in the previous study. For the temporal, prefrontal, occipital and inferior parietal cortices differences between groups are still significant p<0.05. The previously observed trend (p = 0.08 and 0.075) in the cerebellum and thalamus, respectively, now disappeared (p>0.2). The major difference in the results was in the hippocampus, in which previously the difference was 56% (p = 0.004) and in the current work was a 13% difference (p = 0.4).

#### Parkinson’s disease study (PD)

In a recent study using the 2-TCM with ASIF we did not find a significant difference in striatal [^18^F]FEPPA binding in 16 PD subjects (8 HABs) compared to 16 age-matched HC (8 HABs)[8]. PBIF75 was also applied to the same data.

**Fig 6:**
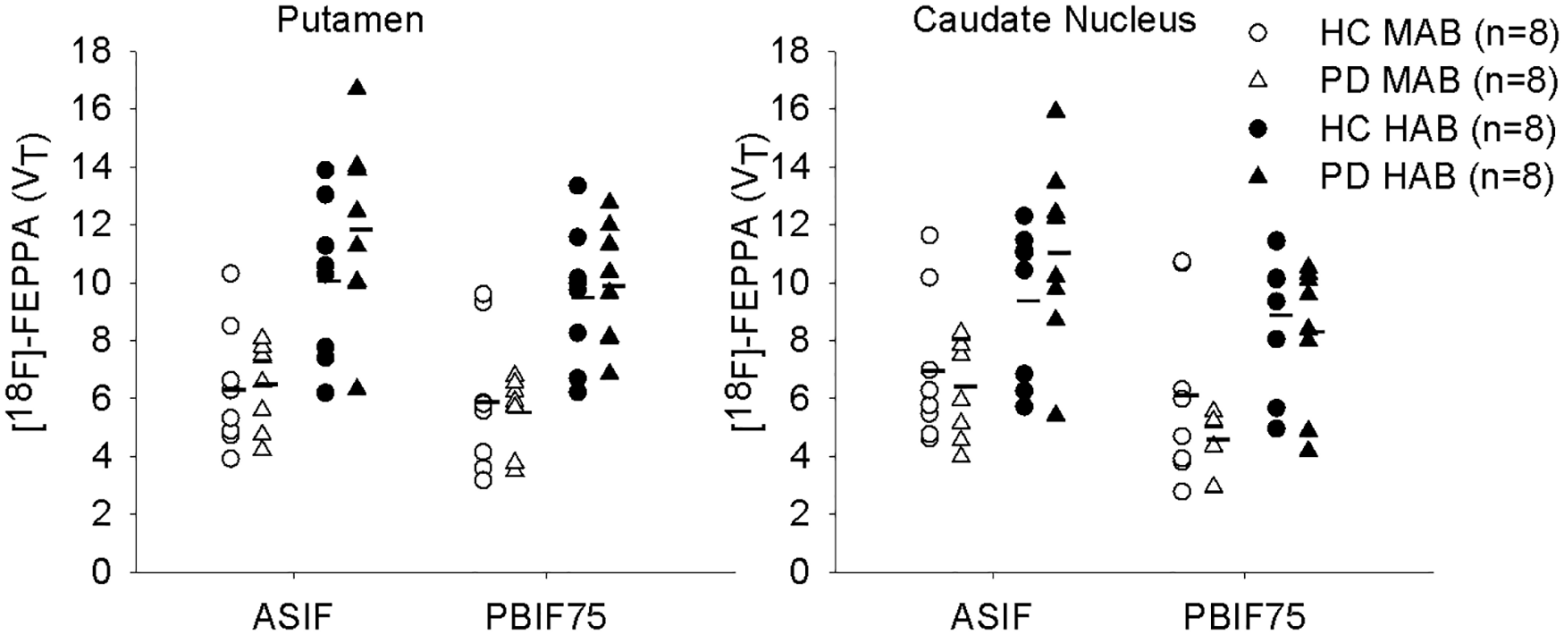
Comparison of total distribution volume (*V*_*T*_) between healthy controls and PD patients calculated with ASIF and PBIF75 for putamen (left) and caudate (right). Estimation with PBIF75 led to same conclusion as with the ASIF; that there is no difference from disease (p = 0.2 in Caudate and p = 0.8 in the Putamen using PBIF vs p = 0.4 in Caudate and p = 0.7 in Putamen using ISIF) and that there is a difference due to genotype (p = 0.001 in Caudate and p = 0.002 in Putamen using PBIF vs p = 0.001 in the Caudate and p = 0.0008 in Putamen using ASIF). Images were partial volume effect corrected prior to TAC extraction.

**Validation of the PBIF75 in PD patients:** It was verified that the optimal sample for rescaling was 75 minutes (Figure E in S1 File) and there was an acceptable agreement between ASIF *V*_*T*_ and PBIF75 for the PD data (caudate ICC = 0.11 for MABs, ICC = 0.6 for HABs and putamen ICC = 0.64 for MABs, ICC = 0.57 for HABs). The decrease in agreement was caused by noisier striatal TACs PVEC in PD subjects, which produced higher variability in 2-TCM and a bias dependent of the magnitude in the Logan estimations. A single data point in each ROI drove the statistical significance of the bias. Consistent with this finding, Fig 6 shows a lower mean *V*_*T*_ for Logan derived PBIF75, especially in the caudate, and a smaller spread in the data with PBIF. Additionally, it was verified that the correlated bias in B&A plots disappears when PBIF is used with 2-TCM (Figure F(Second raw) in S1 File) and still persists when ASIF is applied to both 2-TCM and GA (Figure F(First raw) in S1 File).

***V***_***T***_ **[**^**18**^**F]FEPPA group comparison**: Using PBIF75 we did not find any difference between PD vs HC (ANOVA including genotype as co-variable, putamen p = 0.8 and caudate p = 0.2), which is in agreement with the previously published results using ASIF.

## Discussion

In the present study, we showed that using a scaled average IF, we can reproduce results found previously with the “standard” [^18^F]FEPPA analysis. A sample of concentration of arterial parent compound at 75 minutes contains an important amount of information to individualize the IF. However, it induces an extra variability that translates into a decrease of statistical power. Conversely the detailed knowledge of the individual shape of the input function in [^18^F]FEPPA would help to increase the statistical power.

It has been previously noted that GA is more suited to PBIF than 2-TCM because GA relies on the AUC(t) of the IF and is therefore less sensitive to the shape [20, 30]. The peak heights of PBIF are generally not recovered as accurately as for the tail wherefore the same reasoning extends to PBIF. GA assumes that the linear relation of eq 1 is reached for some t* which depends on the regional tracer kinetics. If t* is chosen too early there is an underestimation of *V*_*T*_, while if it is chosen too late there are fewer points in the linear regression and more variability in *V*_*T*_. An automatic adjustment of t* for each target ROI helps in finding a compromise. We used a 10% admissible error criterion in order to determine the start of the linear behavior of the plot. Ordinary least squared is known to introduce a noise induced underestimation in Logan’s *V*_*T*_[31]. However, since the ROIs considered in this work are quite large (#voxel range from ~ 2554 to ~ 8083) and have TACs that usually do not have a lot of noise, this effect would play a minor role [32]. On other hand, the threshold of 10% was too restrictive in the TACs of the smaller ROIs with low uptake (e.g. caudate head or hippocampus).

The success in the application of the PBIF relies on the temporal evolution of the parent concentration following an undeviating pattern between subjects. We have not seen significant differences in the average IF between clinical groups; however, it is expected that PBIF cannot exactly reproduce the shape of the individual input functions, especially during the early rapid phase as seen in Fig 1A. When the AUC of the PBIF peak is similar to the AUC of ASIF, independently of the shape of the peak, it does not produce a strong impact in the estimation of *V*_*T*_ (Figure H(Left) in S1 File). In contrast, when the mismatch is in the tail (washout period) of the IF (Figure H(Right) in S1 File) the biases in the estimation of *V*_*T*_ are stronger (even when the peak at the early phase is accurately reproduced). This is due to the cumulative difference in the AUC (t) of the IF. In summary, scaling to the tail of the IF is more significant than to the shape of the peak.

We have observed that rescaling the PBIF with either the sample at 60 or at 90 minutes optimizes the difference between AUC of PBIF and the ASIF (Fig 2). However, we chose 75 min, the average between 60 min and 90 min, as the optimal sample. It should be noted that we did not acquire a sample at 75 min, rather it was decided as a way to decrease the impact of errors in the measures of metabolization in our results and to avoid subjectivity in discovering outliers by looking at the pattern of metabolization. Usually the samples of [^18^F]FEPPA at 60 and 90 minutes, when the injected dose is 5 mCi, are reliable enough and not highly susceptible to noise.

Venous sampling at 75 min post-injection was performed on a cohort of subjects who underwent [^18^F]FEPPA PET (n = 8; 5 HABs and 3 MABs) and compared with the average of the arterial samples at 60 and 90 minutes. Our aim was to investigate the possibility of substituting the invasive arterial sample used to calibrate the PBIF by a venous sample. The fraction of unmetabolized radioligand in arterial plasma (Figure I in S1 File) and concentration of radioactivity due to unmetabolized radioligand in arterial plasma (Figure J in S1 File) show an excellent correlation with venous plasma values (r = 0.98,p = 0.00002 and r = 0.93, p = 0.0009, respectively). While in MABs the concentration due to parent compound in venous samples was slightly lower 3%±5%, in HABs there was some underestimation (19%±5%), mostly due to the fraction of unmetabolized [^18^F]FEPPA in plasma (8.5%±3.3% in venous vs. 9.7%±3.6% in arterial, paired t-test p = 0.003). Interestingly, the inter-subjects variability within genotype did not change amongst the two groups. In MABs, CoV = 12% and 14% in arterial and venous samples, respectively; in HABs, CoV = 26% and 29% in arterial and venous samples, respectively. Future studies should include comparisons of venous and arterial samples at other time points, with larger samples and its investigation in clinical populations.

Our results cannot be generalized to other clinical populations without previously verifying the consistency in the input function with respect to the average considered in the present work. In the groups we have studied, we did not observe significant changes in the AUC of the IF. This most likely means that the contribution of factors such as metabolization rate, peripheral binding and blood components binding (*e*.*g* platelets or while cells binding) do not affect the rate of elimination of the radioligand from plasma. For example, we did not observe significant differences in the rate of metabolization between HABs and MABs (Figure G in S1 File). Hence, we were able to build a single PBIF for both HABs and MABs.

The delay between the input function and the TAC usually involves an extra source of variability. In the present work, the delay was estimated with the length of the background frame (frame of variable length between scan acquisition begins and radioactivity appears in the field of view.

It is noteworthy that the measurements of agreement depend on between methods variability in relation to between subject variability. TSPO radioligands are known for presenting a high variability between subjects [33], which could partially benefit the success of the PBIF for this radioligand. Free fraction (protein binding) in plasma is not considered in the analysis; however, it can eventually be applied to the *V*_*T*_ after estimation with either method.

In a previous work[16], we have derived a blood TAC from the image which later was converted to plasma TAC (using the ratio blood to plasma) and corrected by metabolization using 10 manual arterial blood samples (*i*.*e* IDIF). The main purpose of that work was to estimate [^18^F]FEPPA *V*_*T*_ when ABSS was not available. It is very unlikely that the temporal evolution of the ratio blood to plasma and metabolization at early times can be extracted indistinguishable from venous samples. The current work represents a feasibility analysis about the quantification of [^18^F]FEPPA *V*_*T*_ using a single arterial sample at late time point, which eventually can be replaced by a venous sample provided experimental validation. While PBIF is a very simple procedure, IDIF requires a complex algorithm and the availability of the list mode data to do an alternative reconstruction of each image with a particular frame definition, number of iterations and subset of the OSEM-PSF reconstruction. Qualitatively IDIF gives information of the individual peak of the IF that is missing in PBIF. A hybrid between both methods should be studied in the future, as it presents the potential of correcting GA for the vascular contribution and provides more detail on the shape of the IF’s peak

## Conclusion

By using PBIF with Logan graphical analysis and variable t* automatically adjusted with a 10% maximum error criterion to estimate [^18^F]FEPPA *V*_*T*_, we were successful in replicating previous clinical studies of neuroinflamation quantified with 2-TCM and ASIF. Although this technique still requires one arterial blood sample for calibration, it can help when ASIF data is partially missing, or when implementing a quality control on the error prone creation of the IF. If a correlation between radioligand concentration in venous and arterial samples can be established at some time between 60 and 90 minutes, the arterial sample can be replaced by a venous sample, which would simplify the experiment but at the expense of an extra increase of the variability. Caution should be taken when generalizing the results of the present study to other TSPO radiotracers, clinical groups or experiments in which the sample at 75 minutes may not contain all the information needed to account for change in rate of metabolization or radioligand availability.

## Supporting information

S1 File This file contains the supporting information figures. **Figure A. A comparison between IF created with arterial blood samples of healthy controls, patients with Parkinson’s disease and Alzheimer’s disease.** The log transformation was applied on the average of 4 subjects in each genotype dependent group (4 MAB-, 4 HAB-HC; 4 MAB-, 4 HAB-PD and 4 MAB-, 4 HAB-AD subjects respectively). The plot demonstrates that there were no substantial differences in the peak and the tail of the input function between groups. Therefore all subjects were pooled together to create the IF (in red) to be used in the population based method.
**Figure B: [**^**18**^**F]FEPPA V**_**T**_ **estimated by Logan plot overestimated slightly (0.6±1.2 mL/cm3) those estimated with 2-TCM**. The positive bias is consequence of: a) the 2-TCM implementation account for a vascular blood fraction (5%) which was ignored in Logan plot b) the high signal to noise of the big ROIs selected did not induce the characteristic underestimation of Logan method. The 95% limits of agreement (dashed lines) range from -1.3 to 2.6 mL/cm3 and the bias is independent of the V_T_ value. The input function for the kinetic models was computed from arterial blood samples (ASIF) and the plot includes data from 21 HC, 18 AD and 16 PD. MABs and HABs are pool together in the plot.
**Figure C: Bland-Altman plots of regional total distribution volumes (*V***_***T***_**) of healthy controls (4 MABs, 4 HABS) estimated by Logan plot using arterial blood samples (ASIF) and population based input function (popif).** Each plot includes values for the Frontal ctx., Temporal ctx., Striatum, Cerebellar ctx., and Thalamus. From the upper left plot to the lower-right plot, popif was scaled by samples taken at 12, 20, 30, 45, 52, 60, 75, and 90 minutes post injection respectively. Using the 75 minute pseudo-arterial sample to scale popif improves the 95% limits of agreement and does not shown any systematic bias (R^2^ = 0.04, p = 0.05) respect of the V_T_ values.
**Figure D: Bland-Altman plots of regional total distribution volumes (*V***_***T***_**) of Alzheimer’s disease patients (4 HABs, 4 MABs) estimated by Logan plot using arterial blood samples (ASIF) and population based input function (popif).** Each plot includes values for the Frontal ctx., Temporal ctx., Striatum, Cerebellar ctx., and Thalamus. From the upper left plot to the lower-right plot, PBIF was scaled by samples taken at 12, 20, 30, 45, 52, 60, 75, and 90 minutes post injection respectively. Using the 75 minute sample to scale popif improves the 95% limits of agreement and does not shown any systematic bias respect of the V_T_ value.
**Figure E: Bland-Altman plots of regional total distribution volumes (*V***_***T***_**) of Parkinson’s disease patients (4 HABs, 4 MABs) estimated by Logan plot using arterial blood samples (ASIF) and population based input function (popif).** Each plot includes values for the Frontal ctx., Temporal ctx., Striatum, Cerebellar ctx., and Thalamus. From the upper left plot to the lower-right plot, PBIF was scaled by samples taken at 12, 20, 30, 45, 52, 60, 75, and 90 minutes post injection respectively. Using the 75 minute sample to scale popif improves the 95% limits of agreement and does not shown ant systematic bias (R^2^ = 0.03,p = 0.03)respect of the V_T_ value.
**Figure F. Understanding the systematic bias in Bland-Altman plot in Parkinson’s disease patients.** (First raw. A and B): Bland-Altman plot of *V*_*T*_ calculated using ASIF (2TCM) and using PBIF75 (Logan plot) in Parkinson’s disease patients for Caudate and Putamen regions. (A) In MABs (n = 8) the bias is independent of V_T_ (~1.5 ml/cm3)) (B) in HAB (n = 8), the bias has a trend of linear relationship with the magnitude of V_T_ (R^2^ = 0.24, p = 0.1, Putamen and Caudate V_T_ were pooled together to build the model). (Second raw C and D) Bland-Altman plot of *V*_*T*_ derived using ASIF(2TCM) and PBIF75(2TCM) in the Caudate and Putamen regions. (C) MABs (D) HABs. When using 2TCM the bias does not dependent on V_T_ values even when PBIF75 is used. Therefore the correlated bias in B is induced by the Logan plot, and is driven by a single data point in the caudate and a single data point in the putamen.
**Figure G: Metabolization of [^18^F]FEPPA is not different between MABs and HABs**.
**Figure H: Discrepancies between the peak and the washout of the PBIF and ABSS-IF and their effect on the Logan plot slope**. On the left column, when PBIF peaks slightly later than ABSS-IF and with lower maximum activity (A), the area under the curve is not largely different (B) and therefore the slope of Logan plot (*V*_*T*_) is not very different (C). On the right column, when both PBIF and ABSS-IF peak at the same time and value but differ in the washout (D),the area under the curve of both input functions progressively diverged through time (E) and strongly biases the estimation of slope of the Logan plot (*V*_*T*_) (F).
**Figure I**. Correlation of the fraction of unmetabolized parent in arterial plasma (average of arterial samples at 60 and 90 min post-injection) and in venous plasma at 75 min post-injection. Data points are labeled according to each genotype group and were pooled together for the linear regression (line).
**Figure J**. Correlation of the radioactivity concentration due to parent compound in arterial and venous plasma. Data was labeled by genotype. In HABs, venous samples underestimated the concentration given by the arterial samples more than in MABs. High correlations of the samples were found when data was analyzed by genotype (continue lines) and when everything was pooled together (dotted line).
**Table A. Regional [**^**18**^**F]FEPPA VT for AD and healthy control groups.** Factorial ANOVA were performed for each ROI to compare differences between diagnostic groups with genotype and age added as covariates. %Diff was calculated as the difference in [^18^F]FEPPA VT between the groups divided by [^18^F]FEPPA VT of the healthy control group. (*cf*. table2 1 in ref [7]).
(PDF)

S2 File Population based input function. This MS-Excel file contains 24 individual input functions that were used to create the PBIF (also included).
(XLSX)
