# Supplementary material for: Feasibility study of TSPO quantification with [18F]FEPPA using population-based input function
Source: PLoS One. 2017 May 17;12(5):e0177785. doi: 10.1371/journal.pone.0177785 (PMC5435246; doi:10.1371/journal.pone.0177785)
Supplement: S1 File — Figure A. A comparison between IF created with arterial blood samples of healthy controls, patients with Parkinson’s disease and Alzheimer’s disease. The log transformation was applied on the average of 4 subjects in each genotype dependent group (4 MAB-, 4 HAB-HC; 4 MAB-, 4 HAB-PD and 4 MAB-, 4 HAB-AD subjects respectively). The plot demonstrates that there were no substantial differences in the peak and the tail of the input function between groups. Therefore all subjects were pooled together to create the IF (in red) to be used in the population based method. Figure B: [18F]FEPPA VT estimated by Logan plot overestimated slightly (0.6±1.2 mL/cm3) those estimated with 2-TCM. The positive bias is consequence of: a) the 2-TCM implementation account for a vascular blood fraction (5%) which was ignored in Logan plot b) the high signal to noise of the big ROIs selected did not induce the characteristic underestimation of Logan method. The 95% limits of agreement (dashed lines) range from -1.3 to 2.6 mL/cm3 and the bias is independent of the VT value. The input function for the kinetic models was computed from arterial blood samples (ASIF) and the plot includes data from 21 HC, 18 AD and 16 PD. MABs and HABs are pool together in the plot. Figure C: Bland-Altman plots of regional total distribution volumes (VT) of healthy controls (4 MABs, 4 HABS) estimated by Logan plot using arterial blood samples (ASIF) and population based input function (popif). Each plot includes values for the Frontal ctx., Temporal ctx., Striatum, Cerebellar ctx., and Thalamus. From the upper left plot to the lower-right plot, popif was scaled by samples taken at 12, 20, 30, 45, 52, 60, 75, and 90 minutes post injection respectively. Using the 75 minute pseudo-arterial sample to scale popif improves the 95% limits of agreement and does not shown any systematic bias (R2 = 0.04, p = 0.05) respect of the VT values. Figure D: Bland-Altman plots of regional total distribution volumes (VT) of Alzheimer’ [file pone.0177785.s001.pdf]

Population-Based Input Function for TSPO quantification with [^{18}F]FEPPA.

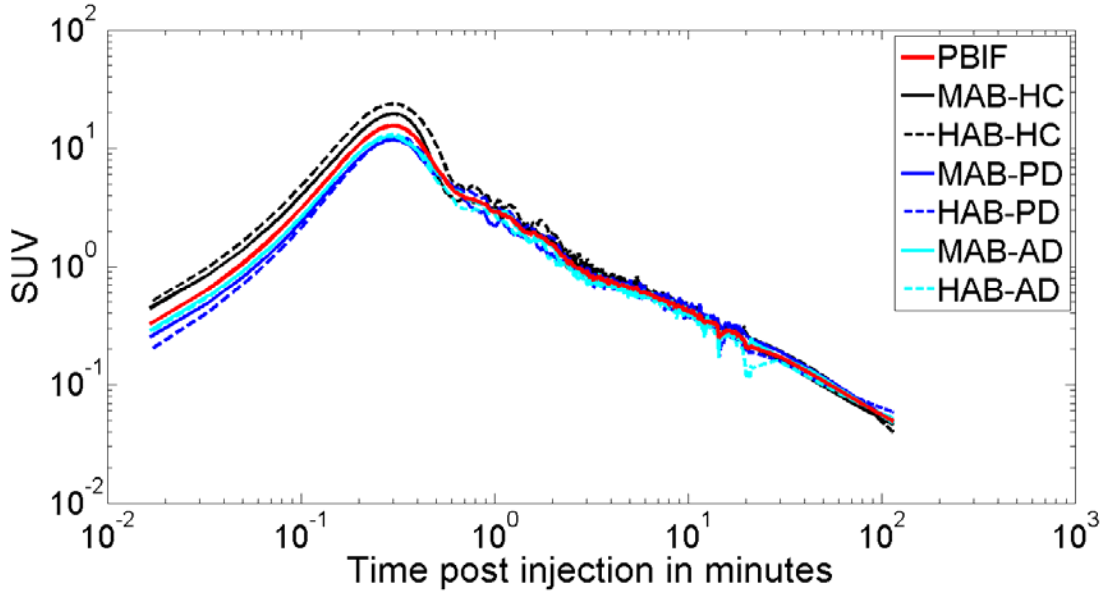

Figure A: A comparison between IF created with blood samples of healthy controls, patients with Parkinson's disease and Alzheimer's disease. The log transformation was applied on the average of 4 subjects in each genotype dependent group (4 MAB-, 4 HAB-HC; 4 MAB-, 4 HAB-PD and 4 MAB-, 4 HAB-AD subjects respectively). The plot demonstrates that there were no substantial differences in the peak and the tail of the input function between groups. Therefore all subjects were pooled together to create the IF (in red) to be used in the population based method.

Population-Based Input Function for TSPO quantification with [^{18}F]FEPPA.

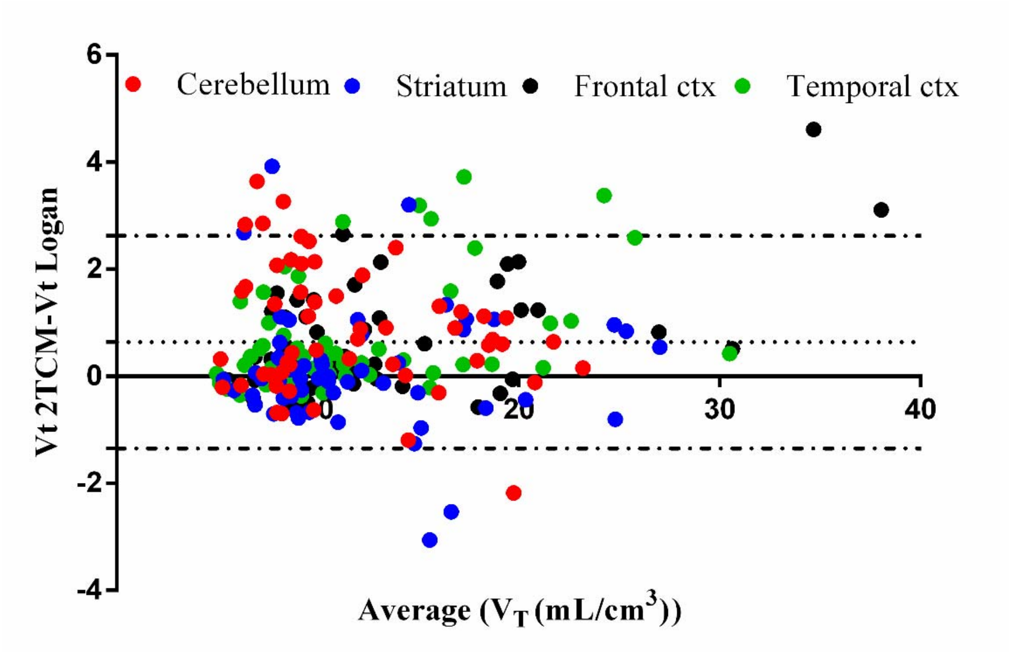

Figure B. [18F]Feppa V_T estimated by Logan plot overestimated slightly (0.6±1.2 mL/cm3) those estimated with 2-TCM. The positive bias is consequence of: a) the 2-TCM implementation account for a vascular blood fraction (5%) which was ignored in Logan plot b) the high signal to noise of the big ROIs selected did not induce the characteristic underestimation of Logan method. The 95% limits of agreement (dashed lines) range from -1.3 to 2.6 mL/cm3 and the bias is independent of the V_T value. The input function for the kinetic models was computed from arterial blood samples (ASIF) and the plot includes data from 21 HC, 18 AD and 16 PD. MABs and HABs are pool together in the plot.

Population-Based Input Function for TSPO quantification with [^{18}F]FEPPA.

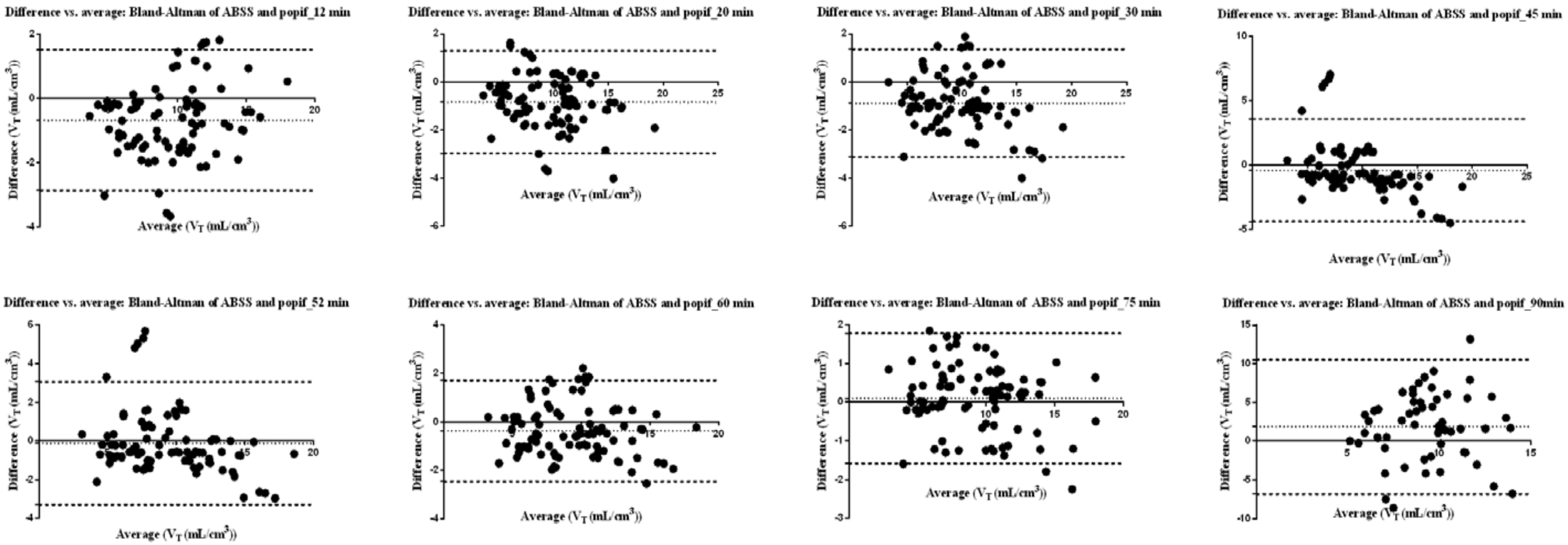

Figure C: Bland-Altman plots of regional total distribution volumes (V_T) of healthy controls (4 MABs, 4 HABS) estimated by Logan plot using arterial blood samples (ASIF) and population based input function (popif). Each plot includes values for the Frontal ctx. , Temporal ctx., Striatum, Cerebellar ctx., and Thalamus. From the upper left plot to the lower-right plot, popif was scaled by samples taken at 12, 20, 30, 45, 52, 60, 75, and 90 minutes post injection respectively. Using the 75 minute pseudo-arterial sample to scale popif improves the 95% limits of agreement and does not shown any systematic bias (R^2=0.04, p=0.05) respect of the V_T values.

Supplementary Material

Population-Based Input Function for TSPO quantification with $[^{18}F]$FEPPA.

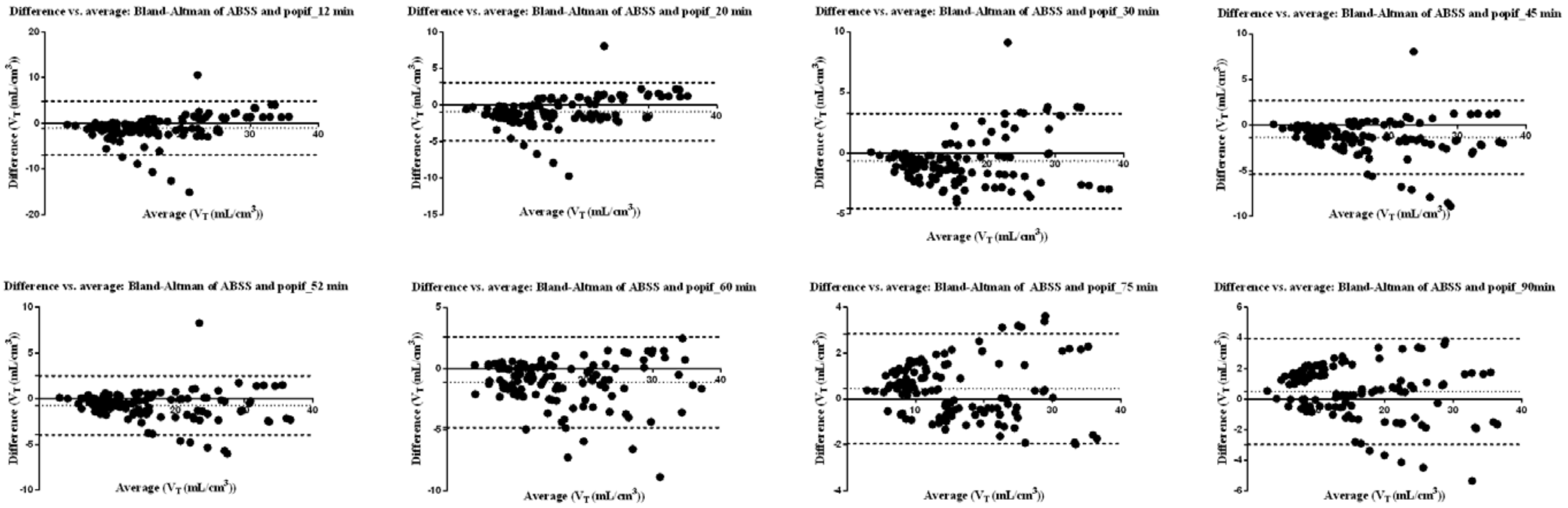

Figure D: Bland-Altman plots of regional total distribution volumes (V_T) of Alzheimer's disease patients (4 HABs, 4 MABs) estimated by Logan plot using arterial blood samples (ASIF) and population based input function (popif). Each plot includes values for the Frontal ctx. , Temporal ctx., Striatum, Cerebellar ctx., and Thalamus. From the upper left plot to the lower-right plot, PBIF was scaled by samples taken at 12, 20, 30, 45, 52, 60, 75, and 90 minutes post injection respectively. Using the 75 minute sample to scale popif improves the 95% limits of agreement and does not shown any systematic bias respect of the V_T value.

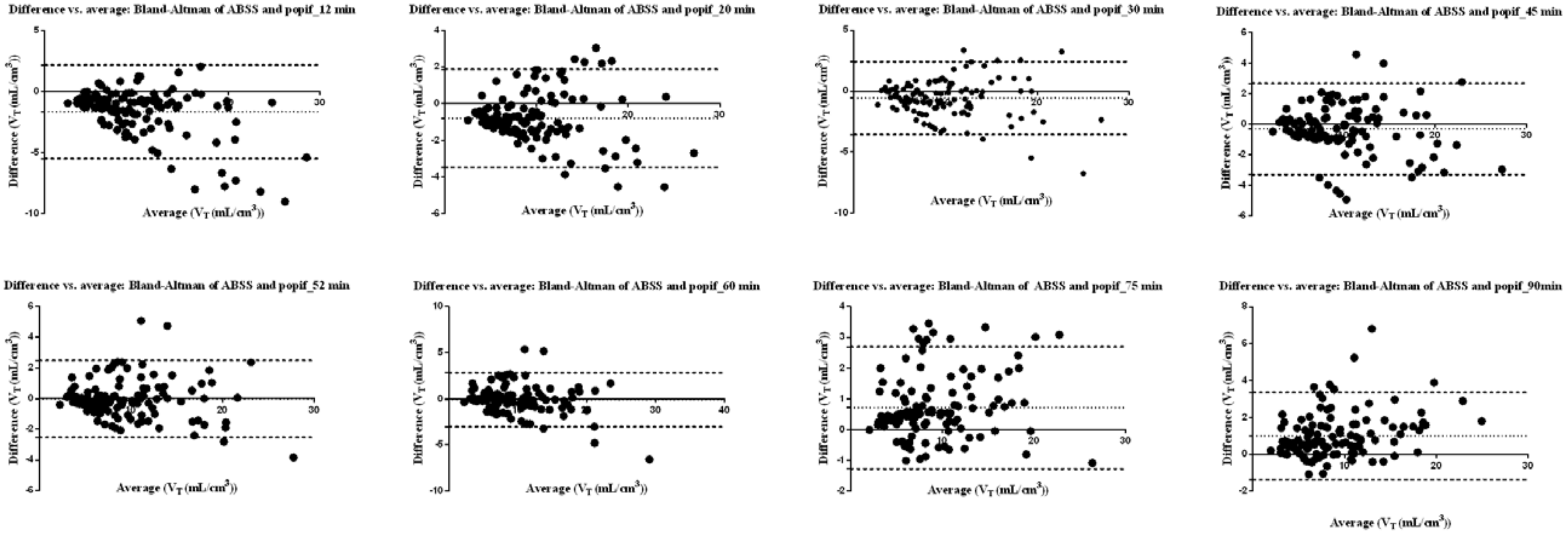

Figure E: Bland-Altman plots of regional total distribution volumes (V_T) of Parkinson's disease patients (4 HABs, 4 MABs) estimated by Logan plot using arterial blood samples (ASIF) and population based input function (popif). Each plot includes values for the Frontal ctx. , Temporal ctx., Striatum, Cerebellar ctx., and Thalamus. From the upper left plot to the lower-right plot, PBIF was scaled by samples taken at 12, 20, 30, 45, 52, 60, 75, and 90 minutes post injection respectively. Using the 75 minute sample to scale popif improves the 95% limits of agreement and does not shown ant systematic bias (R^2=0.03,p=0.03)respect of the V_T value.

Population-Based Input Function for TSPO quantification with [^{18}F]FEPPA.

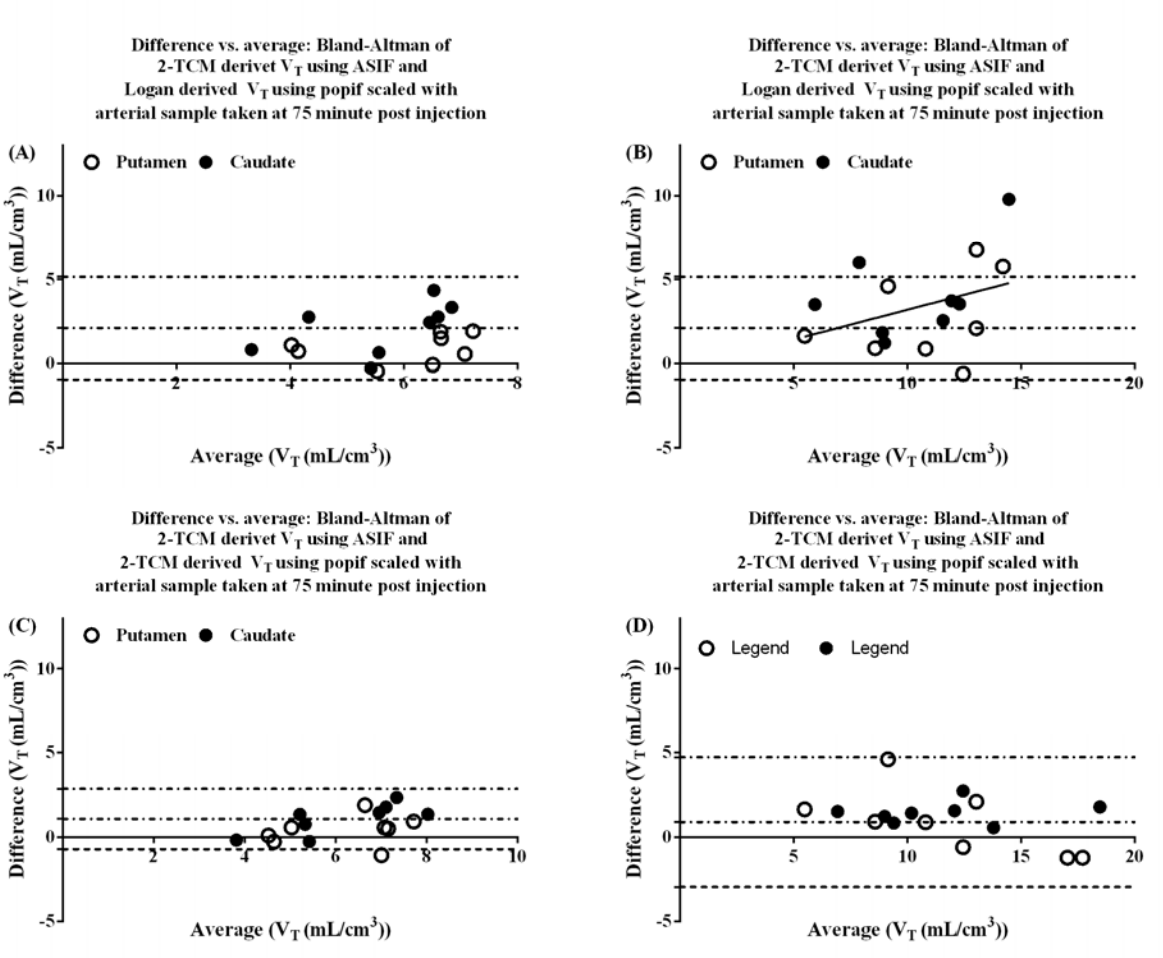

Figure F (First raw. A and B): Bland-Altman plot of V_T calculated using ASIF (2TCM) and using PBIF75 (Logan plot) in Parkinson's disease patients for Caudate and Putamen regions. (A) In MABs (n=8) the bias is independent of V_T (~1.5 ml/cm3)) (B) in HAB (n=8), the bias has a trend of linear relationship with the magnitude of V_T (R^2=0.24, p=0.1, Putamen and Caudate V_T were pooled together to build the model). (Second raw C and D) Bland-Altman plot of V_T derived using ASIF(2TCM) and PBIF75(2TCM) in the Caudate and Putamen regions. (C) MABs (D) HABs. When using 2TCM the bias does not dependent on V_T values even when PBIF75 is used. Therefore the correlated bias in B is induced by the Logan plot, and is driven by a single data point in the caudate and a single data point in the putamen.

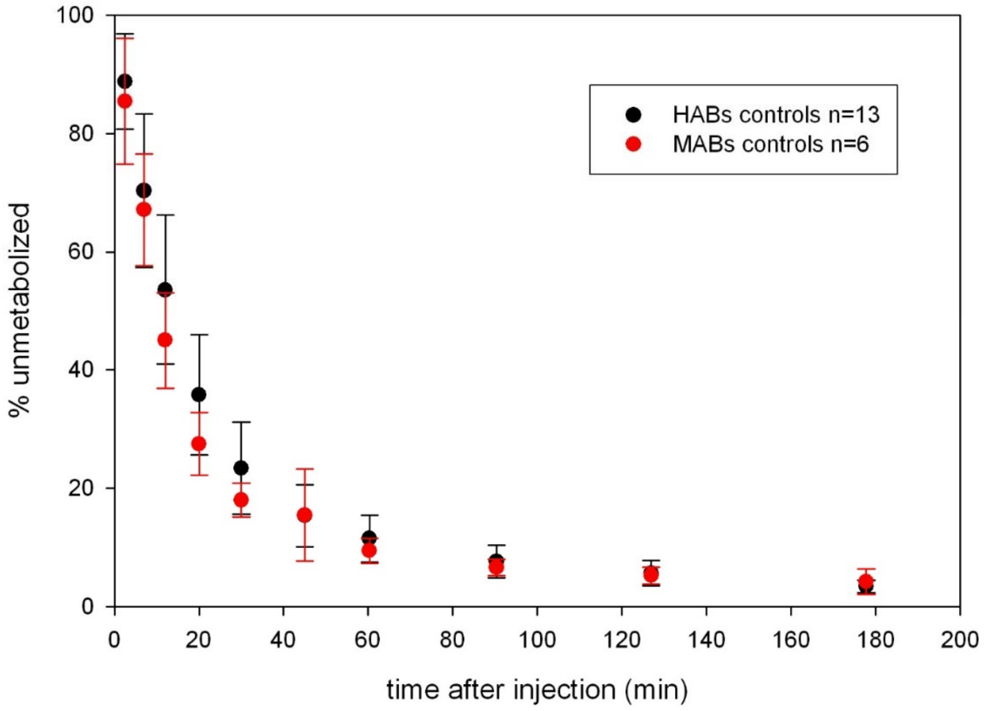

Figure G: Metabolization of [^{18}F]-FEPPA is not different between MABs and HABs.

Population-Based Input Function for TSPO quantification with [^{18}F]FEPPA.

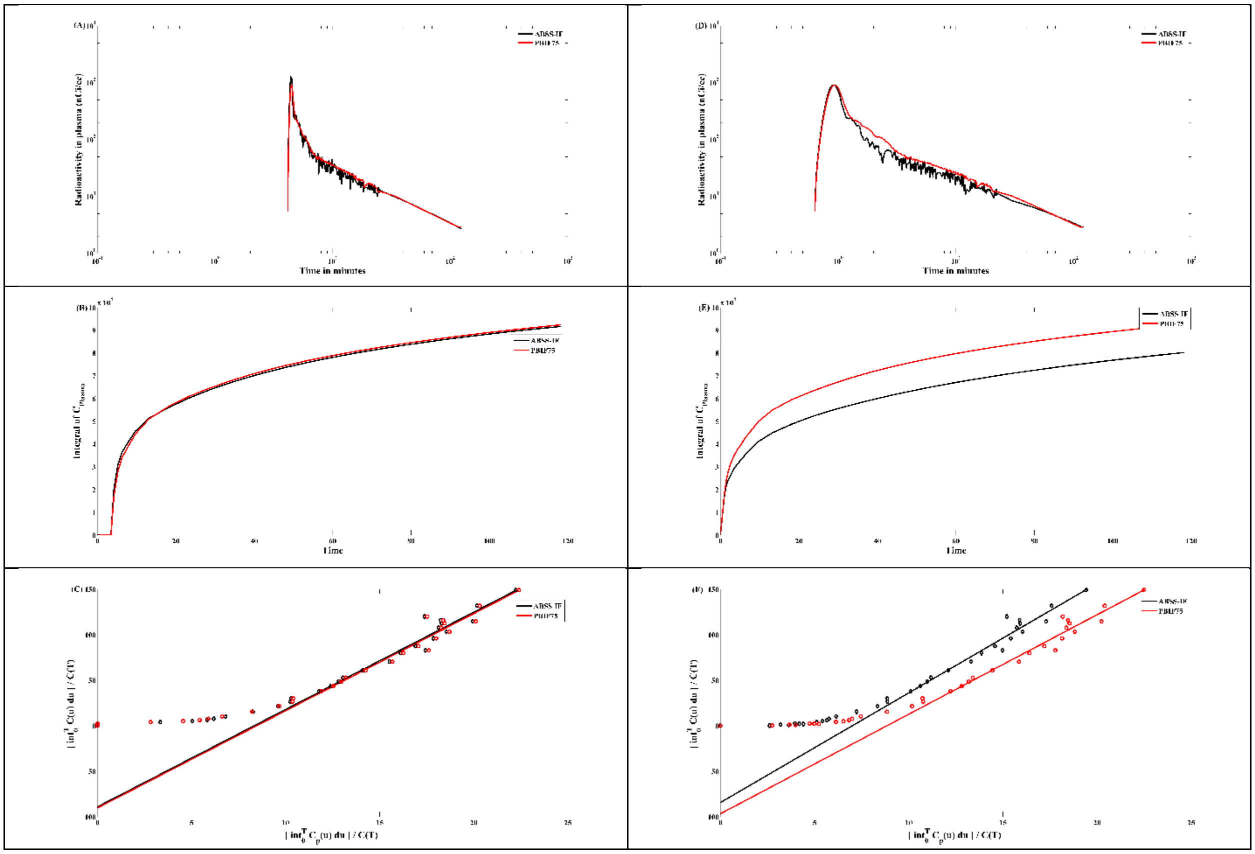

Figure H: Discrepancies between the peak and the washout of the PBIF and ABSS-IF and their effect on the Logan plot slope. On the left column, when PBIF peaks slightly later than ABSS-IF and with lower maximum activity (A), the area under the curve is not largely different (B) and therefore the slope of Logan plot (V_T) is not very different (C). On the right column, when both PBIF and ABSS-IF peak at the same time and value but differ in the washout (D),the area under the curve of both input functions progressively diverged through time (E) and strongly biases the estimation of slope of the Logan plot (V_T) (F).

Population-Based Input Function for TSPO quantification with [^{18}F]FEPPA.

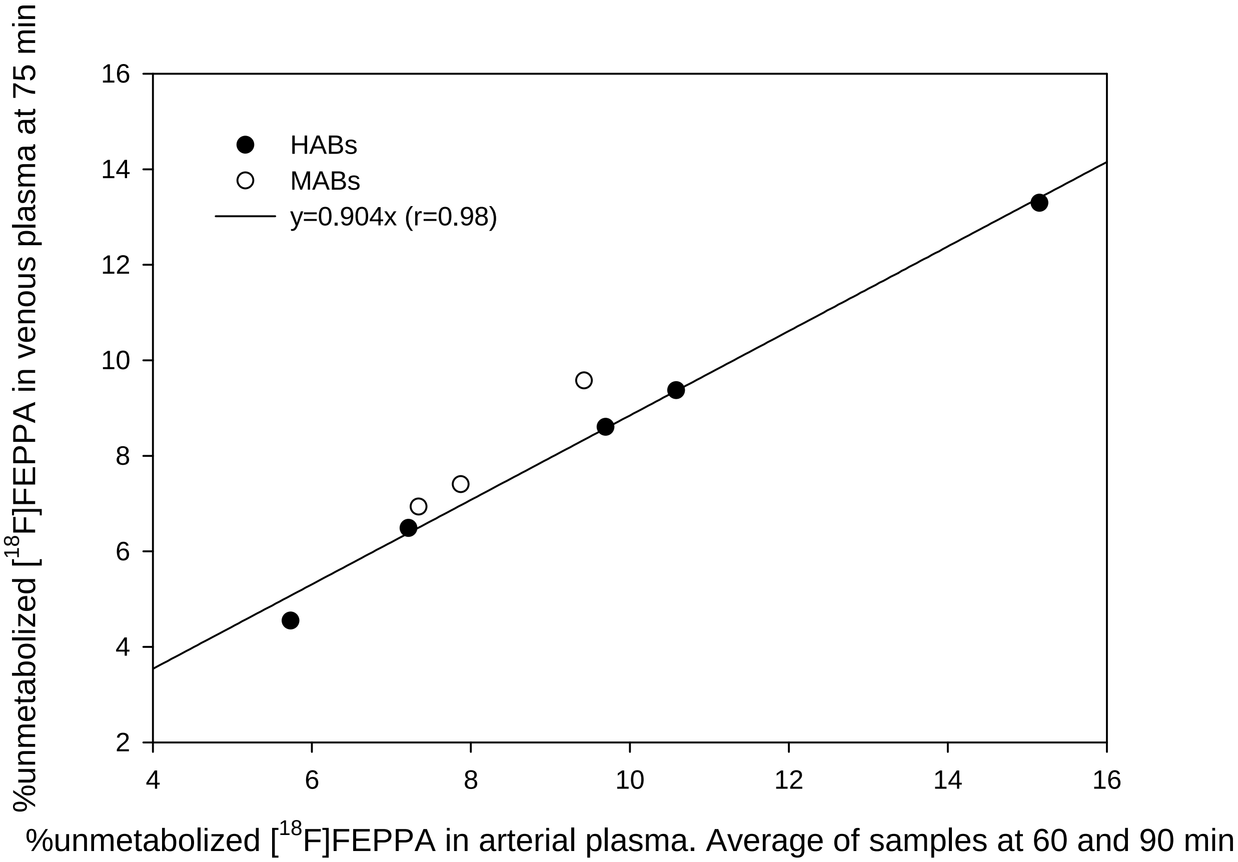

Figure I. Correlation of the fraction of unmetabolized parent in arterial plasma (average of arterial samples at 60 and 90 min post-injection) and in venous plasma at 75 min post-injection. Data points are labeled according to each genotype group and were pooled together for the linear regression (line).

Population-Based Input Function for TSPO quantification with $[^{18}F]$FEPPA.

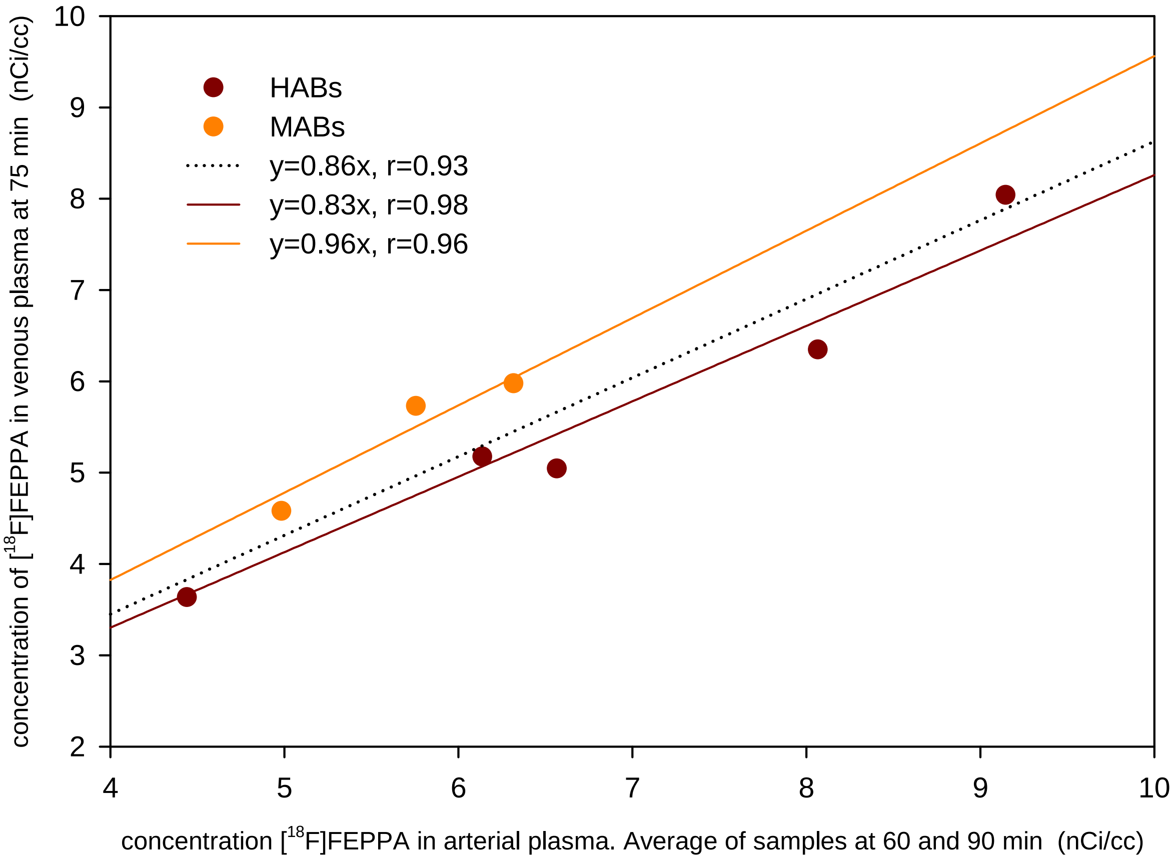

Figure J. Correlation of the radioactivity concentration due to parent compound in arterial and venous plasma. Data was labeled by genotype. In HABs, venous samples underestimated the concentration given by the arterial samples more than in MABs. High correlations of the samples were found when data was analyzed by genotype (continue lines) and when everything was pooled together (dotted line).

Supplementary Material

Population-Based Input Function for TSPO quantification with [^{18}F]FEPPA.

	Heathy Controls (n=21)		Alzheimer's Disease (n=18)			Diagnostic	
ROI	adj mean	std err	adj mean	std err	%Diff	F (1,35)	p
Temporal ctx	12.073	1.284	16.387	1.393	36%	4.878	.034
PreFrontal ctx	14.071	1.322	20.393	1.435	45%	9.876	.003
Inf_parietal ctx	13.986	1.380	19.841	1.498	42%	7.774	.009
Occipital ctx	11.588	1.506	17.104	1.635	48%	5.793	.022
Hippocampus	9.471	1.038	10.686	1.127	13%	.591	.447
Cerebellar ctx	10.382	.905	12.086	.982	16%	1.532	.224
Thalamus	11.891	1.172	13.559	1.272	14%	.875	.356

Table A. Regional [^{18}F] FEPPA VT for AD and healthy control groups. Factorial ANOVA were performed for each ROI to compare differences between diagnostic groups with genotype and age added as covariates. %Diff was calculated as the difference in [^{18}F]FEPPA VT between the groups divided by [^{18}F]FEPPA VT of the healthy control group. (*cf.* table2 1 in ref [1])

References

1. Suridjan I, Pollock BG, Verhoeff NP, Voineskos AN, Chow T, Rusjan PM, et al. In-vivo imaging of grey and white matter neuroinflammation in Alzheimer's disease: a positron emission tomography study with a novel radioligand, [18F]-FEPPA. Molecular psychiatry. 2015;20(12):1579-87. doi: 10.1038/mp.2015.1. PubMed PMID: 25707397.
